# Complete Recovery With the Chain of Survival After a Prolonged (120 Minutes) Out-of-Hospital Cardiac Arrest Due to Brugada Syndrome: A Case Report

**DOI:** 10.1097/MD.0000000000001107

**Published:** 2015-07-13

**Authors:** Fei He, Peng Xu, Zhong-Hai Wei, Jun Zhang, Jun Wang

**Affiliations:** From the Department of Emergency Medicine (FH, PX, JZ, JW); Department of Cardiology (Z-HW), Nanjing Drum Tower Hospital, Nanjing University Medical School, Nanjing, China.

## Abstract

Out-of-hospital cardiac arrest (OHCA) is a crucial public health problem. To improve outcomes of patients after cardiac arrest, the American Heart Association promotes the concept of the chain of survival.

We report a case of a 19-year-old man with no markedly past medical history who suffered from OHCA, and he was resuscitated with cardiopulmonary resuscitation, without interruption, during the rescue process for 120 minutes until return of spontaneous circulation (ROSC). Electrocardiogram on admission showed right bundle branch block and ST segment elevation in leads V1–V2, and the patient's uncle had experienced the same event and had received implantable cardioverter defibrillator (ICD) treatment. Therefore, the patient was diagnosed with Brugada syndrome. Postcardiac arrest care was performed after ROSC, including mild therapeutic hypothermia, hemodynamic monitoring and management, and ICD implantation, and then the patient completely recovered without any noticeable neurological or intellectual deficits in the follow-up examinations.

Our case demonstrates that even after an OHCA with prolonged time (120 minutes) until ROSC, survival with a favorable neurological outcome is possible, provided implementation of an extremely effective rescue chain.

## INTRODUCTION

Brugada syndrome is an autosomal dominant genetic heart disease.^1^ It is thought to be responsible for fatal ventricular tachyarrhythmia that may lead to syncope or out-of-hospital cardiac arrest (OHCA) in young people without structural heart disease.^2,3^ Because arrhythmic events are more common at night and during sleep, it is difficult to diagnose and treat patients with Brugada syndrome.^4^ Here, we report a case of OHCA caused by Brugada syndrome, which had been successfully treated with “chain of survival” interventions.

## CASE REPORT

A 19-year-old man with no markedly past medical history was found lying on the floor, unresponsive, by his colleagues at 09:40 AM of October 28, 2014. One colleague immediately called the emergency medical services (EMSs) by mobile phone, while another performed bystander cardiopulmonary resuscitation (CPR). The ambulance with 2 EMS providers arrived within 5 minutes. They found the patient pulseless, with no signs of breathing and nonreacting pupils. The first electrocardiogram detected pulseless electrical activity (PEA). The patient was transported to the emergency department under continuous manual chest compression. The patient did not receive any medications before admission.

**FIGURE 1:**
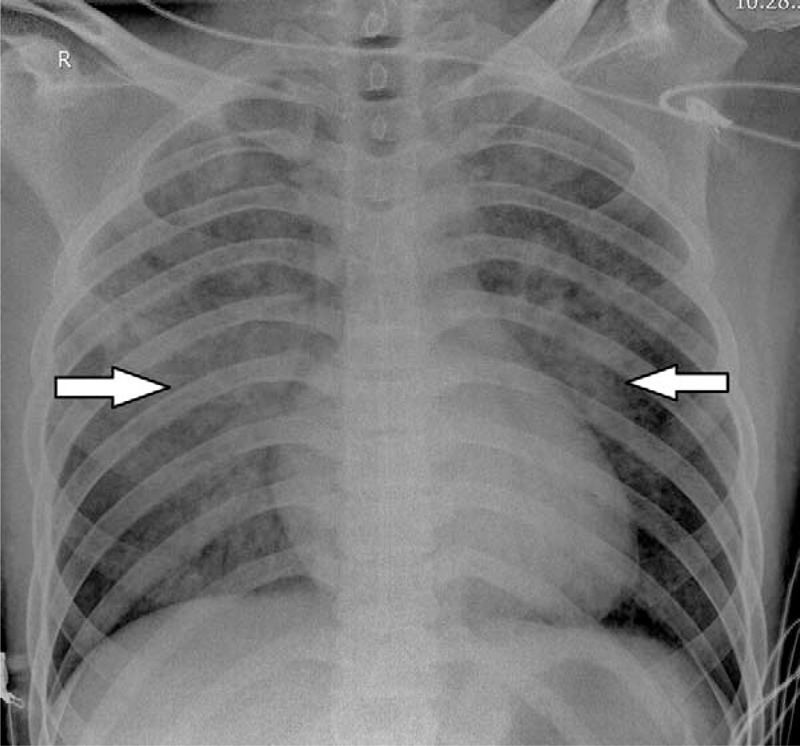
Chest x-ray in 1 hour after ROSC showed a hazy opacification spreading from the lung hilar regions (arrows). ROSC = return of spontaneous circulation.

The patient arrived at the Emergency Department of Nanjing Drum Tower Hospital 15 minutes after cardiac arrest. He had poor general conditions with Glasgow Coma Scale score 3; he had no spontaneous respirations and heart rhythm, no response to verbal stimuli, and had fixed and dilated pupils. The advanced life support protocol was immediately started by emergency physicians in our department. The patient was intubated and mechanically ventilated, while CPR was continuously performed using a gas-driven mechanical chest compression (Grand Rapids, Michigan Instruments; Hirtz, Koln, Germany) with compression rate 100/min and deepness of chest compression 5 cm). Ventricular fibrillation was first detected 35 minutes after cardiac arrest by the defibrillator, and 4 defibrillations (ventricular fibrillation or pulseless ventricular tachycardia) were performed in all, following injections of adrenaline (5 mg in all), as well as amiodarone and dopamine infusions. One hundred and twenty minutes after cardiac arrest, he presented successful return of spontaneous circulation (ROSC) with blood pressure 85/42 mm Hg and heart rate 103 bpm. The patient showed hypoxaemia (pulse oxygen saturation <90%; positive end-expiratory pressure 12 mm Hg, Fio_2_ 100%, 1 hour after ROSC), although invasive mechanical ventilation was given. The arterial blood gas was pH 7.309, partial arterial CO_2_ pressure 40.3 mm Hg, PO2 52.8 mm Hg, Fio_2_ 100%, and Po_2_/Fio_2_ 52.8, and the chest x-ray showed pulmonary edema (Figure 1).

**FIGURE 2:**
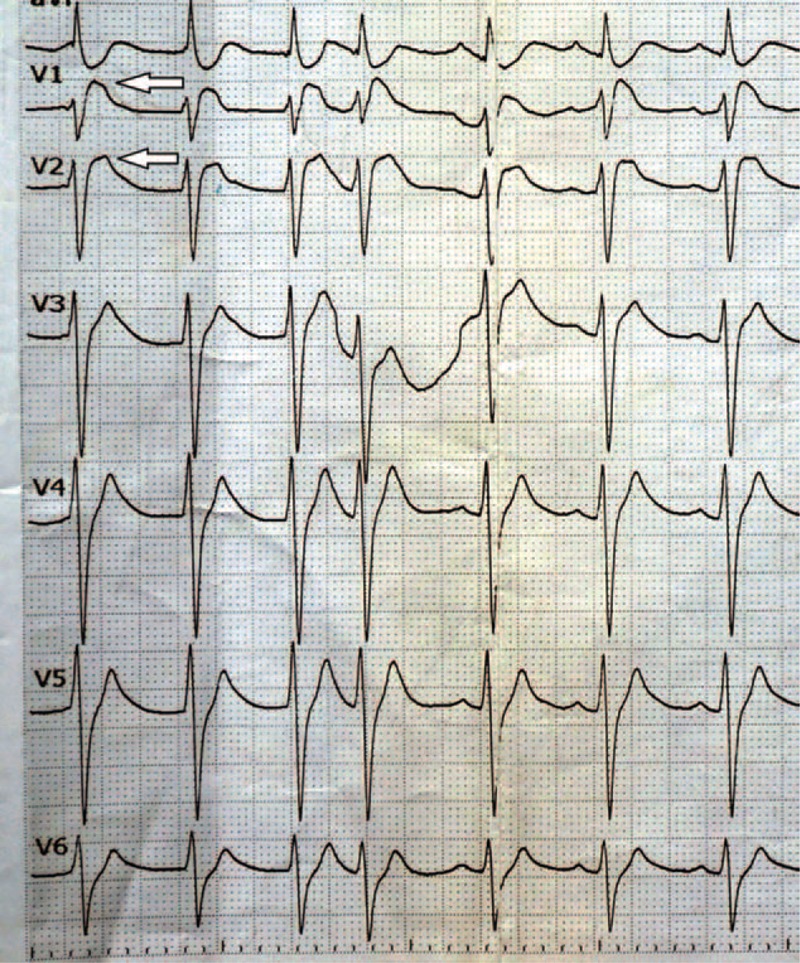
Electrocardiograms in precordial leads after ROSC showed right bundle branch block and ST segment elevation in leads V1–V2 (arrows). ROSC = return of spontaneous circulation.

Then the patient was moved to the emergency intensive care unit 3.5 hour after the onset, and his vital signs were as follows: temperature 39°C, heart rate 110 bpm, blood pressure 83/55 mm Hg (dopamine infusion 12 mcg/kg · min), and oxygen saturation 95% (mechanical ventilation, Fio_2_ 50%), and the serum neuron-specific enolase (NSE) was significantly elevated (75.05 ng/mL, reference 0–16.3 ng/mL). Neurological examination was notable for the following: disappeared spontaneous respiration, fixed and dilated pupils, absent corneal reflexes, and absent motor responses to painful stimuli in all extremities. The repeat electrocardiogram showed a right bundle branch block and ST segment elevation in leads V1–V2 (Figure 2). The patient's uncle had experienced the same event and had received implantable cardioverter defibrillator (ICD) treatment 1 year earlier. Therefore, the patient was diagnosed with Brugada syndrome.

A mild therapeutic hypothermia (MTH) was started (∼5 hours after cardiac arrest) by infusing a cold (4°C) saline solution (1.8 L in all). The body temperature decreased to 35.9°C (24 hours after cardiac arrest), and the core temperature was maintained between 34°C and 36°C for 24 hours in combination with an external cooling mattress (HICO-Hypotherm 680 of Hirtz and HICO-Polyurethan water mat; Hirtz, Koln, Germany). Shivering appeared in MTH; the shivering was suppressed with lytic cocktail therapy (chlorpromazine, promethazine, and pethidine) and high doses of sedatives (midazolam, fentanyl, and propofol). No other complications occurred during the cooling procedure. Bedside echocardiography showed left heart insufficiency and an ejection fraction of 33%. Because of heart dysfunction and unstable circulation (systolic blood pressure 76–83 mm Hg), the patient received dopamine and an intraaortic balloon pump (IABP), and pulse index continuous cardiac output (PiCCO) monitoring system was introduced (Table 1). Active rewarming was initiated at 0.2°C/hour 48 hours after cardiac arrest. The rewarming goal temperature of 37.0°C was reached within 6 hours. Dopamine infusion and IABP were then discontinued because circulatory condition and heart function had improved.

**TABLE 1:**
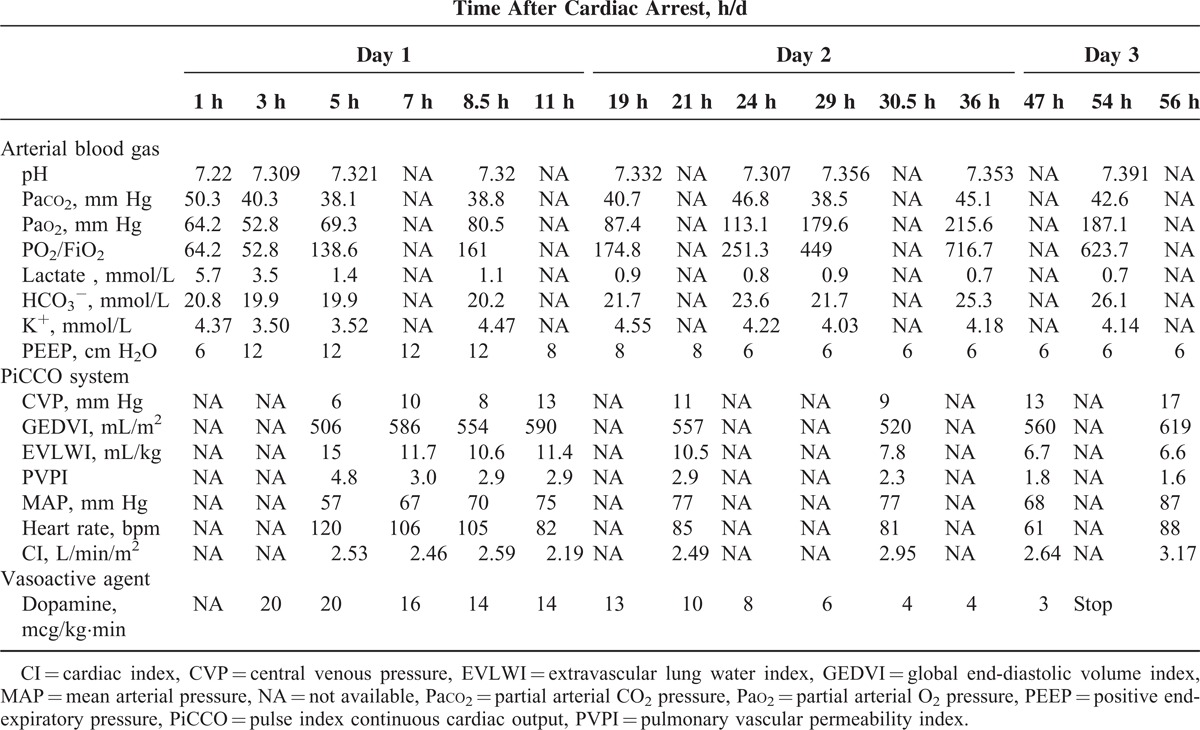
Time Course and Data

**FIGURE 3:**
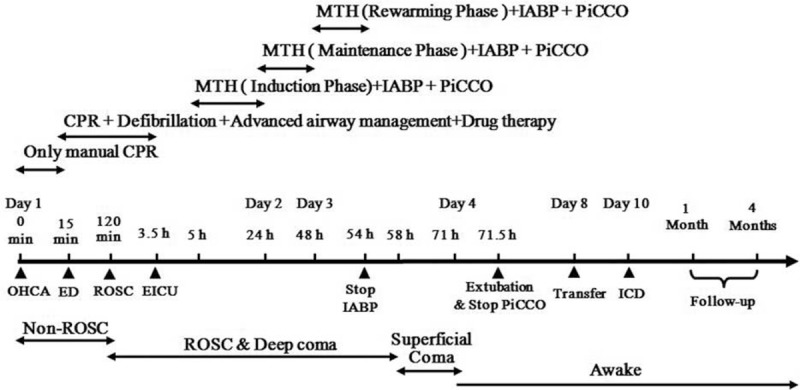
Course of the patient after OHCA caused by Brugada syndrome. CPR = cardiopulmonary resuscitation, ED = emergency department, EICU = emergency intensive care unit, IABP = intraaortic balloon pump, ICD = implantable cardioverter defibrillator, MTH = mild therapeutic hypothermia, OHCA = out-of-hospital cardiac arrest, PiCCO = pulse index continuous cardiac output, ROSC = return of spontaneous circulation.

The patient's consciousness improved 58 hours after cardiac arrest; he was able to open his eyes to command and respond to painful stimuli. He was awake and intermittently moved his extremities 71 hours after cardiac arrest. The patient was extubated after a successful spontaneous breathing trial on day 4 after cardiac arrest. Then he spoke in full sentences, was fully aware of person, place, and time, and accurately exhibited full strength in all his extremities. The repeat NSE was 13.77 ng/mL. A noncontrast head computed tomography scan was performed, which showed no evidence of intracranial hemorrhage, mass infarction, or cerebral edema. He was able to ambulate with a walker and independently perform activities of daily life on day 5 after the cardiac arrest. Magnetic resonance imaging and an electroencephalogram were performed, showing nothing abnormal. He completely recovered with 1 point based on the criteria of cerebral performance category^5^ on day 8 after cardiac arrest and was then transferred to the cardiology department. He was implanted an ICD on day 10 after the cardiac arrest. The patient was followed up at 1 and 4 months after discharge and he had no noticeable neurological or intellectual deficits (Figure 3).

## DISCUSSION

OHCA is a crucial public health problem. The estimated incidence of EMS-treated OHCA in the United States is about 40.3 to 86.7/100 000 persons/year, and the overall survival rate is only 3.0% to 16.3%.^6^ To improve outcomes of patients after cardiac arrest, the American Heart Association promotes the concept of the chain of survival.^7^ Following the new principle, patients with OHCA have a higher survival rate and better neurological outcomes.^8,9^

Basic life support (BLS) is the foundation of chain of survival for a higher survival rate.^10^ Continuous chest compression is a critical component of BLS because cardiac output and coronary perfusion depend on compression during CPR.^11^ Therefore, chest compressions should be the highest priority and the initial action for the patients with OHCA. In our case, high-quality CPR that contributed to the successful resuscitation and favorable neurological outcome was performed, without interruption, during the rescue process until ROSC. Although the treatment of mechanical chest compression–decompression system in OHCA is controversial,^12,13^ at least during prolonged resuscitation, as in our case, it may avoid reducing the quality of CPR. Zimmermann et al^14^ also found that mechanical CPR improved the outcome of a patient with OHCA.

Early defibrillation is critical to survival from OHCA because the most frequent initial heart rhythm in OHCA is ventricular fibrillation, which should be immediately defibrillated.^15^ However, no defibrillation was performed out of hospital in our case because the initial monitored heart rhythm of the patient was PEA, and bystander-performed CPR is more important than defibrillation for patients with a nonshockable rhythm.^16^

Postcardiac arrest care has significant potential to reduce early mortality caused by hemodynamic instability, and later mortality and morbidity from multiorgan failure and neurological function damage.^17,18^ MTH, as a critical component of postcardiac arrest care, is an important resuscitation treatment that improves neurological outcomes and gives a better chance of survival to discharge in survivors with cardiac arrest.^19,20^ Our case also showed that MTH after ROSC greatly improved the neurological outcome in the patient. A difference between our case and earlier studies is that our patient was maintained with a temperature between 34°C and 36°C.^19,20^ Although therapeutic hypothermia (targeted temperature from 32°C to 34°C) is now recommended in international resuscitation guidelines,^21^ a recent international multicenter trial demonstrates that a targeted temperature of 36°C has comparable survival rate and neurological function of OHCA patients, compared with the temperature of 33°C.^22^

Hyperthermia should be avoided following OHCA because it is associated with worse outcome.^23^ However, shivering is common and may raise body temperature when inducing MTH. Failure to control shivering is a common reason for delays in achieving target temperatures.^24,25^ Our patient was persistently shivering after ROSC and early phrase of MTH; it took us long time with sedation to control and achieve the target temperature.

The complex hemodynamic instability, including hypovolemia, myocardial stunning, excessive vasodilation, and pulmonary edema, is common after ROSC, which is associated with increased mortality.^26^ Therefore, it is important to monitor and evaluate hemodynamic variables in patients after ROSC under normothermic and hypothermic conditions. In our case, hypotension, heart dysfunction, and pulmonary edema occurred soon after ROSC and during the cooling procedure. We monitored cardiopulmonary function and hemodynamic parameters using the PiCCO monitoring system (Table 1), and improved the cardiopulmonary function using IABP and diuretics, all of these measures are very important in goal-directed fluid management patients after OHCA.

The diagnosis and treatment of the cause of underlying disease is fundamental to the management of OHCA. Brugada syndrome is diagnosed in our case with typical electrocardiogram findings (coved ST elevation in leads V1–V2) and family history. An operation of ICD implantation was successfully performed for the patient, and he showed complete recovery in the follow-up examinations.

In conclusion, our case demonstrates that even after an OHCA with prolonged time until ROSC, survival with a favorable neurological outcome is possible, provided implementation of an extremely effective rescue chain.

## References

[1] Sheikh AS Ranjan K Brugada syndrome: a review of the literature. *Clin Med* 2014; 14:482–489. 25301907 10.7861/clinmedicine.14-5-482 PMC4951955

[2] Sacher F Arsac F Wilton SB Syncope in Brugada syndrome patients: prevalence, characteristics, and outcome. *Heart Rhythm* 2012; 9:1272–1279. 22504046 10.1016/j.hrthm.2012.04.013

[3] Antzelevitch C Brugada P Borggrefe M Brugada syndrome: report of the second consensus conference: endorsed by the Heart Rhythm Society and the European Heart Rhythm Association. *Circulation* 2005; 111:659–670. 15655131 10.1161/01.CIR.0000152479.54298.51

[4] Atarashi H Ogawa S Harumi K Characteristics of patients with right bundle branch block and ST-segment elevation in right precordial leads. Idiopathic Ventricular Fibrillation Investigators. *Am J Cardiol* 1996; 78:581–583. 8806350 10.1016/s0002-9149(96)00360-8

[5] Jennett B Bond M Assessment of outcome after severe brain damage. *Lancet* 1975; 1:480–484. 46957 10.1016/s0140-6736(75)92830-5

[6] Nichol G Thomas E Callaway CW Regional variation in out-of-hospital cardiac arrest incidence and outcome. *JAMA* 2008; 300:1423–1431. 18812533 10.1001/jama.300.12.1423 PMC3187919

[7] Travers AH Rea TD Bobrow BJ Part 4: CPR Overview: 2010 American Heart Association Guidelines for Cardiopulmonary Resuscitation and Emergency Cardiovascular Care. *Circulation* 2010; 122:S676–684. 20956220 10.1161/CIRCULATIONAHA.110.970913

[8] Rea TD Eisenberg MS Sinibaldi G Incidence of EMS treated out-of-hospital cardiac arrest in the United States. *Resuscitation* 2004; 63:17–24. 15451582 10.1016/j.resuscitation.2004.03.025

[9] Cummins RO Eisenberg MS Prehospital cardiopulmonary resuscitation. Is it effective? *JAMA* 1985; 253:2408–2412. 3981769

[10] Berg RA Hemphill R Abella BS Part 5: Adult Basic Life Support: 2010 American Heart Association Guidelines for Cardiopulmonary Resuscitation and Emergency Cardiovascular Care. *Circulation* 2010; 122:S685–S705. 20956221 10.1161/CIRCULATIONAHA.110.970939

[11] Tang CL Cheung KS Tsui SH Successful resuscitation after out-of-hospital cardiac arrest. *Hong Kong Med J* 2012; 18:536–538. 23223658

[12] Lafuente-Lafuente C Melero-Bascones M Active chest compression–decompression for cardiopulmonary resuscitation. *Cochrane Database Syst Rev* 2004; 2:CD002751. 15106176 10.1002/14651858.CD002751.pub2

[13] Nielsen N Sandhall L Scherstén F Successful resuscitation with mechanical CPR, therapeutic hypothermia and coronary intervention during manual CPR after out-of-hospital cardiac arrest. *Resuscitation* 2005; 65:111–113. 15797284 10.1016/j.resuscitation.2004.11.007

[14] Zimmermann S Rohde D Marwan M Complete recovery after out-of-hospital cardiac arrest with prolonged (59 min) mechanical cardiopulmonary resuscitation, mild therapeutic hypothermia and complex percutaneous coronary intervention for ST-elevation myocardial infarction. *Heart Lung* 2014; 43:62–65. 24238746 10.1016/j.hrtlng.2013.10.011

[15] Link MS Atkins DL Passman RS Part 6: electrical therapies: automated external defibrillators, defibrillation, cardioversion, and pacing: 2010 American Heart Association Guidelines for Cardiopulmonary Resuscitation and Emergency Cardiovascular Care. *Circulation* 2010; 122:S706–719. 20956222 10.1161/CIRCULATIONAHA.110.970954

[16] Kuo CW See LC Tu HT Adult out-of-hospital cardiac arrest based on chain of survival in Taoyuan county, northern Taiwan. *J Emerg Med* 2014; 46:782–790. 24094529 10.1016/j.jemermed.2013.08.026

[17] Nolan JP Neumar RW Adrie C Post-cardiac arrest syndrome: epidemiology, pathophysiology, treatment, and prognostication: a Scientific Statement from the International Liaison Committee on Resuscitation; the American Heart Association Emergency Cardiovascular Care Committee; the Council on Cardiovascular Surgery and Anesthesia; the Council on Cardiopulmonary, Perioperative, and Critical Care; the Council on Clinical Cardiology; the Council on Stroke. *Resuscitation* 2008; 79:350–379. 18963350 10.1016/j.resuscitation.2008.09.017

[18] Carr BG Kahn JM Merchant RM Inter-hospital variability in post-cardiac arrest mortality. *Resuscitation* 2009; 80:30–34. 18952359 10.1016/j.resuscitation.2008.09.001

[19] Hypothermia after Cardiac Arrest Study Group. Mild therapeutic hypothermia to improve the neurologic outcome after cardiac arrest. *N Engl J Med* 2002; 346:549–556. 11856793 10.1056/NEJMoa012689

[20] Bernard SA Gray TW Buist MD Treatment of comatose survivors of out-of-hospital cardiac arrest with induced hypothermia. *N Engl J Med* 2002; 346:557–563. 11856794 10.1056/NEJMoa003289

[21] Peberdy MA Callaway CW Neumar RW Part 9: Post-Cardiac Arrest Care: 2010 American Heart Association Guidelines for Cardiopulmonary Resuscitation and Emergency Cardiovascular Care. *Circulation* 2010; 122:S768–S786. 20956225 10.1161/CIRCULATIONAHA.110.971002

[22] Nielsen N Wetterslev J Cronberg T Targeted temperature management at 33°C versus 36°C after cardiac arrest. *N Engl J Med* 2013; 369:2197–2206. 24237006 10.1056/NEJMoa1310519

[23] Gebhardt K Guyette FX Doshi AA Prevalence and effect of fever on outcome following resuscitation from cardiac arrest. *Resuscitation* 2013; 84:1062–1067. 23619740 10.1016/j.resuscitation.2013.03.038

[24] Badjatia N Strongilis E Gordon E Metabolic impact of shivering during therapeutic temperature modulation: the Bedside Shivering Assessment Scale. *Stroke* 2008; 39:3242–3247. 18927450 10.1161/STROKEAHA.108.523654

[25] Hostler D Northington WE Callaway CW High-dose diazepam facilitates core cooling during cold saline infusion in healthy volunteers. *Appl Physiol Nutr Metab* 2009; 34:582–586. 19767791 10.1139/H09-011

[26] Tagami T Kushimoto S Tosa R The precision of PiCCO^®^ measurements in hypothermic post-cardiac arrest patients. *Anaesthesia* 2012; 67:236–243. 22321078 10.1111/j.1365-2044.2011.06981.x

